# Carotenoids and Fatty Acids Obtained from Paprika *Capsicum annuum* by Supercritical Carbon Dioxide and Ethanol as Co-Extractant

**DOI:** 10.3390/molecules28145438

**Published:** 2023-07-16

**Authors:** Dorota Kostrzewa, Barbara Mazurek, Marcin Kostrzewa, Emilia Jóźwik

**Affiliations:** 1 Łukasiewicz Research Network—New Chemical Syntheses Institute, Aleja Tysiąclecia Państwa Polskiego 13A, 24-110 Pulawy, Poland; barbara.mazurek@ins.lukasiewicz.gov.pl (B.M.); emilia.jozwik@ins.lukasiewicz.gov.pl (E.J.); 2 Faculty of Chemical Engineering and Commodity Science, Kazimierz Pulaski University of Technology and Humanities in Radom, Chrobrego 27, 26-600 Radom, Poland; m.kostrzewa@uthrad.pl

**Keywords:** *Capsicum annuum*, carotenoids, polyunsaturated fatty acids, supercritical carbon dioxide extraction, co-extractant

## Abstract

Paprika *Capsicum annuum* L. contains useful molecules such as carotenoids and polyunsaturated fatty acids, which are considered high-value functional and health ingredients. To obtain these compounds, paprika was extracted using different methods (Soxhlet, SC-CO_2,_ and SC-CO_2_ with co-extractant) and at different parameters. The results showed that the carotenoid content decreased with the addition of the co-extractant while the fatty acid content and yield increased. It was found that the highest carotenoid content (capsanthin > β-carotene > capsorubin > zeaxanthin > β-cryptoxanthin > violaxanthin) was obtained at 50 °C/45 MPa for SC-CO_2_ extraction. Paprika extract rich in polyunsaturated fatty acids (linoleic, oleic, and α-linolenic acid) was obtained at 40 °C/25 MPa for SC-CO_2_ with co-extractant. The PUFA/SFA ratios for paprika extract were in agreement with the recommendations of nutritional guidelines. The use of SC-CO_2_ for the extraction of Capsicum annuum allowed us to obtain a high-quality, rich in carotenoids and polyunsaturated fatty acids, extract that can be used as a substrate in the industry.

## 1. Introduction

In recent times, there has been growing interest in the characterization of products obtained from natural plant materials that are capable of providing additional benefits to human health. In this respect, paprika *Capsicum annuum* L. offers great potential as a source for the extraction of substances with desirable properties [1]. Paprika contains an abundance of minerals, vitamins, polyphenols, and tocopherol, as well as carotenoids and polyunsaturated fatty acids. Based on the components present in this matrix, its products can be potentially used as additives in food, cosmetic and pharmaceutical formulations [2,3,4,5].

Carotenoids present in paprika are desirable compounds since they exhibit excellent antioxidant activity and significant anti-inflammatory benefits [5,6,7]. These natural colorants have the potential to protect against different chronic diseases such as cardiovascular diseases, type 2 diabetes, and atherosclerosis [8]. They strengthen the immune system and reduce the risk of cataracts, gastric ulcers, age-related macular degeneration, and other degenerative diseases [9,10]. The major carotenoid in red paprika, capsanthin, is effective as a free radical scavenger and has a positive effect on the cholesterol level in plasma [11]. It shows a significant effect of neutralization of singlet oxygen and inhibiting effect on lipid peroxidation [12]. Capsanthin exhibits skin photo-protective, antidiabetic, antiobesity, antiadipogenic, and antihyperlipidemic activities [13,14,15,16]. Additionally, capsanthin, β-cryptoxanthin, zeaxanthin, and β-carotene are the carotenoids of *Capsicum* that have been reviewed for their proven anticarcinogenic effects with potential uses in cancer chemoprevention [7].

Paprika *Capsicum* is also a source rich in polyunsaturated fatty acids (PUFAs) [17]. These PUFAs are in the whole fruit and seeds, but their seeds show even greater concentration per gram [18]. PUFAs and specifically linoleic acid and α-linolenic acid present in paprika, are recognized as essential fatty acids. They are precursors of other important fatty acids in metabolism such as arachidonic acid and eicosapentaenoic acid or prostaglandins [19]. PUFAs provide health benefits because they reduce total cholesterol and body fat [4]. Research evidence suggests that n-3 PUFAs are cardioprotective, perhaps through their anti-inflammatory, antiarrhythmic, lipid-lowering, and antihypertensive effects [19]. Humans are not able to synthesize essential fatty acids and can only obtain them through their diet.

Linoleic acid is worth mentioning because this fatty acid may have favorable nutritional implications and beneficial physiological effects in the prevention of both coronary heart disease and cancer [4,18,20]. Other unsaturated fatty acids such as α-linolenic and oleic acids are also important because of their health-promoting properties. The high content of α-linolenic acids contributes to the enhancement of the nutritional value of the obtained product [21], while oleic acid can help reduce the risk of heart diseases by raising levels of high-density lipoprotein and lowering low-density lipoprotein [22]. Reports show that a saturated fatty acid such as palmitic acid present in paprika offers the appropriate amount of plasticity for soap manufacturing and may provide an advantage in paint and coating formulation [18].

Supercritical carbon dioxide (SC-CO_2_) extraction has been developed for over forty years. The process is applied in research as well as for industrial purposes [23]. On a large industrial scale, typical applications are the extraction of hop components, decaffeination of tea and coffee, and the separation of lecithin from oil [24]. SC-CO_2_ is an interesting alternative to the traditional organic solvent extraction method because it provides a non-toxic, efficient, and faster extraction process. A clear advantage is that carbon dioxide is a GRAS (Generally Recognized as Safe) solvent with low critical parameters and it is chemically inert, non-explosive, and easily removable from the final product. The solubility of a selected group of compounds and the selectivity of the process can be increased by applying various extraction parameters. However, components with high molecular weight and polarity have low solubility in carbon dioxide, and changes in temperature and pressure have a limited effect on the extraction process of these compounds. Another way to increase the solubility of a substance is to add a small amount of a carrier agent with GRAS status, such as water, ethanol, or vegetable oils. This substance can be introduced into the carbon dioxide stream (co-solvent) or directly mixed with a plant matrix before the extraction (co-extractant). However, the use of co-solvent requires a personal pump to operate and dissolve in SC-CO_2_ before entering the extraction vessel. Whereas the extraction of SC-CO_2_ with a co-extractant could solve the problem of using a tremendous amount of hazardous organic solvent and eliminate the disadvantages of co-solvent use. This method has been successfully used to obtain bioactive compounds from natural plants [25,26,27].

Although there are some studies of the supercritical extraction of red paprika in the literature, there is scarce information about the qualitative and quantitative composition of carotenoid compounds in the paprika extract. The majority just report the total content of carotenoids. Jimenez et al. [28] and Barros et al. [29] reported only the capsanthin and capsorubin content in paprika extract, while Tepić et al. [21] reported also β-cryptoxanthin and β-carotene content, and Uquiche et al. [30] also zeaxanthin content. To the best of our knowledge, the effect of SC-CO_2_ extraction parameters on violaxanthin content in the paprika extract was not reported. Additionally, the evaluation of the fatty acid content in the paprika extract is also still incomplete. Only a few experiments with fatty acid composition have been done with paprika products [17,18,21]. Most of them concern the extraction of fatty acids from paprika seeds. Moreover, the fatty acid yield for paprika extraction and the effects of co-extractant on individual carotenoids and fatty acid content in the extract was not reported.

Paprika *Capsicum annuum* is a source of substances with desirable properties. It consists of high content and unique carotenoid composition (high content of the paprika-specific oxocarotenoids capsanthin and capsorubin) in combination with a high level of polyunsaturated fatty acids. However, there is an absence of a comparison report of the quality of products obtained using pure SC-CO_2_ and SC-CO_2_ with ethanol as co-extractant in the literature. In this paper, we assessed the effect of ethanol addition and SC-CO_2_ extraction parameters on the yield and content of individual carotenoids and fatty acids in the extract obtained from *Capsicum annuum*. The obtained results provide valuable information for other researchers and companies interested in the processing of paprika for different industrial applications. They can also be useful for transferring the experiment to a larger scale. The results of SC-CO_2_ extraction were compared to those of n-hexane extraction.

## 2. Results and Discussion

### 2.1. The Extraction of Individual Carotenoids Using SC-CO_2_

In the pure SC-CO_2_, extract yield ranged from 7.22 g of extract/100 g dry weight of raw material to 9.94 g/100 g dry weight of the sample. The highest yield was achieved at 35 MPa/60 °C/60 min, while the lowest yield values were achieved at 25 MPa/50 °C/10 min. The SC-CO_2_ extract yield in the present work was lower than the yield achieved by Daood et al. [31] and Tepić et al. [21] and higher than the yield achieved by Gnayfeed et al. [32] and Illés et al. [33]. The analysis of the impact of parameters on SC-CO_2_ paprika extraction yield was carried out in previous works, so it was not presented here [34].

Table 1 contains the carotenoid profile of extracts obtained by SC-CO_2_ extraction under different conditions (SFE1-SFE15) and the Soxhlet extraction using n-hexane (SOX). Violaxanthin, capsorubin, capsanthin, zeaxanthin, β-cryptoxanthin and β-carotene were found in most of the extracts tested. Violaxanthin was not detected only in the extract obtained at 25 MPa/50 °C/10 min (SFE12). Results analysis show that the major carotenoid contained in the paprika extract was capsanthin, which is compliant with the literature reports presented by Kim et al. and Aizawa and Inakuma [11,35]. Capsanthin content in the extract obtained using SC-CO_2_ ranged from 1720 to 18,610 mg/kg extract. Violaxanthin was the carotenoid present at the lowest concentrations in most of the extracts tested, with values ranging from 60 (SFE6) to 750 mg/kg extract (SFE5). The content of individual carotenoids in SC-CO_2_ extracts and their participation changed depending on the extraction parameters applied.

The highest capsanthin content in the extract was obtained at 45 MPa/50 °C/60 min (SFE5). The mass of capsanthin in this extract constituted 64% of the mass of all carotenoids. Then, the lowest mass participation of β-carotene and β-cryptoxanthin was noticed (14.79% and 3.34%, respectively). For the discussed experiment, the highest content of capsorubin > zeaxanthin > β-cryptoxanthin > violaxanthin was also achieved. Extract with the lowest content of violaxanthin, capsanthin, zeaxanthin, β-cryptoxanthin, and β-carotene was obtained at 25 MPa/50 °C/10 min. Then, the mass participation of β-carotene was the highest (50.16%). The results also showed that SC-CO_2_ paprika extract obtained at 45 MPa/50 °C/60 was characterized by a higher content of selected carotenoids than n-hexane paprika extract.

The maximum capsanthin content obtained in the present work was higher than the capsanthin content achieved by Barros et al. (11 mg/g) [29], Tepić et al. (9.204 mg/g) [21] and Uquiche et al. (0.91 mg/g) [30] and was lower only than capsanthin content achieved by Jimenez et al. (148 mg/g) [28]. The maximum capsorubin content obtained in the present work was higher than the capsorubin content reported by Jimenez et al. (0.83 mg/g) [28], Uquiche et al. (1.1 mg/g) [30], and Tepić et al. (0.564 mg/g) [21]. In addition, the maximum content of β-cryptoxanthin and β-carotene in the extract obtained in the present work was 4.18- and 1.34-fold higher than that reported by Tepić et al. [21], whereas 1.09- and 7.20-fold higher than reported by Uquiche et al. [30], respectively. However, the maximum zeaxanthin content in the extract reported only by Uquiche et al. [30] was 1,75-fold higher than in the extract obtained in the present work. Tepić et al. [21] not detected zeaxanthin and violaxanthin in the paprika extract obtained with SC-CO_2_ extraction. This discrepancy in the result may be associated with differences in the chemical composition of the raw material, preparing of raw material for extraction, and process parameters used in each study. Dry paprika extraction, higher extraction pressure, and consumption of CO_2_ contributed to the higher zeaxanthin content in the extract obtained by Uquiche et al. [30]. In the case of the extract obtained by Jimenez et al. [28], the high capsanthin content was caused by the use of dry paprika without seeds and the low oil content in raw material. In previous studies, it was shown that moisture content and the content of lipid compounds in raw material affect the recovery of carotenoids and carotenoid content in the extract [36]. The higher content of water in raw materials has a negative impact on the recovery of carotenoids and a large amount of extracted lipid compounds can lead to dilution of the carotenoids concentration.

Based on the experimental data presented in Table 1, the statistical analysis of the second-order polynomial equation for the content of violaxanthin, capsorubin, capsanthin, zeaxanthin, and β-cryptoxanthin was carried out and regression coefficients were determined. As can be seen in Table 2, the *p*-value for each model was less than 0.05. This means that the full quadratic models are statistically significant. The determination coefficient R^2^ for each model was higher than 0.9, indicating a high correlation between input and output data. At the same time, the analysis showed that some terms of the equations are statistically insignificant (*p* > 0.10). Moreover, the difference between predicted R^2^ and adjusted R^2^ for some responses is larger than recommended one (>0.2) and the lack of fit is significant. Therefore, the model reduction was effected in the subsequent stage using a step-by-step method. The results of the statistical analysis for reduced mathematical models are presented in Table 3. The study showed that the content of β-carotene in paprika extract did not depend statistically significantly on the pressure, temperature, and extraction time, as well as the interaction of these parameters within the range of variables studied. Therefore, they are not included in Table 2 and Table 3.

The reduction in statistically insignificant factors led to the significant improvement of predicted R^2^ for the studied models. The value of predicted R^2^ of reduced models for violaxanthin, capsorubin, capsanthin, zeaxanthin and β-cryptoxanthin increased to 0.8129, 0.7825, 0.9410, 0.9560 and 0.6149, respectively. The predicted R^2^ for each model was in good agreement with the adjusted R^2^. The reduced quadratic models are highly statistically significant (*p* < 0.0005) and the lack of fit is statistically insignificant (*p* > 0.05). It confirmed that the reduced quadratic models can be used to predict values of response variables in the studied range of variability of independent variables.

It can be concluded from the statistical analysis of regression coefficients that the content of violaxanthin, capsanthin, and zeaxanthin in paprika extract depends linearly on the pressure (X_1_), temperature (X_2_), and extraction time (X_3_). Moreover, significant terms of the models include quadratic terms of time (X_3_^2^), whereas interactions of the independent variables (X_1_X_2_, X_1_X_3_, X_2_X_3_), quadratic term of pressure (X_1_^2^), and temperature (X_2_^2^) were regarded as statistically insignificant (*p* > 0.10). For the concentration of capsorubin in the extract, significant terms of the model are linear terms of pressure (X_1_), temperature (X_2_), and time (X_3_), the interaction between pressure and temperature (X_1_X_2_*)* as well as between pressure and time (X_1_X_3_*),* quadratic terms of pressure (X_1_^2^) and quadratic terms of time (X_3_^2^). For the concentration of β-cryptoxanthin in the extract, significant terms of the model are linear terms of pressure (X_1_), temperature (X_2_), time (X_3_), and interaction between pressure and time (X_1_X_3_*).* These results indicated that the effect of various factors on the content of carotenoids was not a simple linear relationship. The final reduced mathematical models for response variables in the coded form are given below.

C_violaxanthin_ = 258.57 + 175.00X_1_ + 48.75X_2_ + 143.75X_3_ + 67.68X_3_^2^ (1)

C_capsorubin_ = 1259.23 + 652.50X_1_ + 147.50X_2_ + 582.50X_3_ + 155.00X_1_X_2_ + 315.00X_1_X_3_ + 173.85X_1_^2^ + 228.85X_3_^2^ (2)

C_capsanthin_ = 8221.43 + 4197.50X_1_ + 807.50X_2_ + 3970.00X_3_ + 1236.07X_3_^2^ (3)

C_zeaxanthin_ = 657.14 + 295.00X_1_ + 85.00X_2_ + 287.50X_3_ + 72.86X_3_^2^ (4)

C_β-cryptoxanthin_ = 700.00 + 142.50X_1_ + 90.00X_2_ + 145.00X_3_ − 90.00X_1_X_3_ (5)

**Figure 1:**
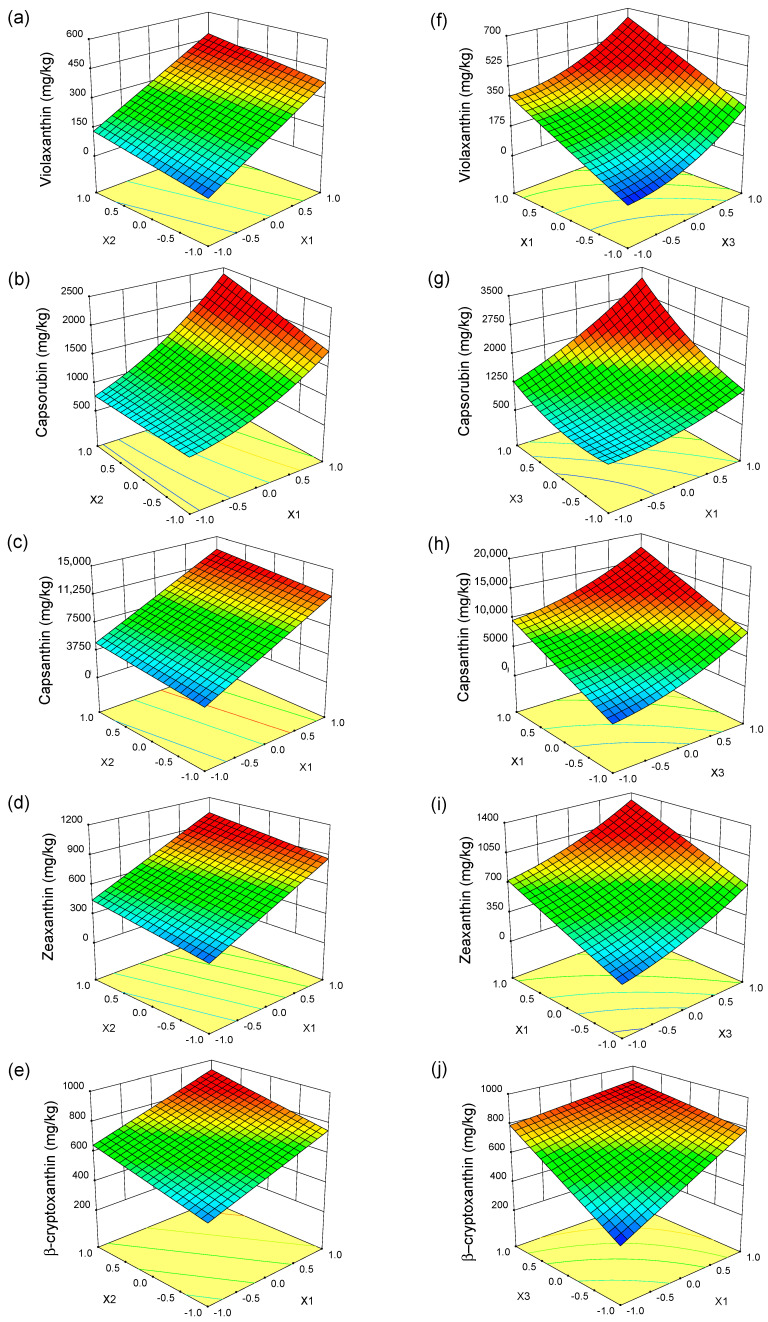
Response surface plots for the content of individual carotenoids in the extract as a function of (**a**–**e**) pressure (X_1_) and temperature (X_2_); (**f**–**j**) pressure (X_1_) and time (X_3_).

The effects of process parameters on the content of violaxanthin, capsorubin, capsanthin, zeaxanthin, and β-cryptoxanthin in the paprika extract are presented as response surface diagrams (Figure 1). As seen in Figure 1a–e, the content of carotenoids (capsanthin, zeaxanthin, violaxanthin, β-cryptoxanthin, and capsorubin) in the extract increased with increasing pressure at a constant temperature. The main reason was the increase in the density of the solvent and its solvation power leading to an increase in the solubility of the carotenoids. Moreover, with increasing density, the molecular distance decreases, and the molecular interactions between the solvent and the extract increase. Additionally, higher pressures may disrupt the other stronger chemical interactions between different compounds and the plant cell wall structures, which may facilitate the release of carotenoids from the extraction bed [37,38]. As a result of increasing the extraction pressure from 25 MPa to 45 MPa at 50 °C, the content of violaxanthin in the extract increased 6.5 fold, the content of capsanthin, capsorubin and zeaxanthin increased almost 3 fold and the content of β-cryptoxanthin increased nearly 2 fold. A positive impact of pressure on carotenoid solubility was also reported by Jarén-Galán et al. [39] and Tepić et al. [21].

The carotenoid content also increased with increasing temperature at a given pressure despite the decrease in carbon dioxide density (Figure 1a–e). It can be explained by the increased volatility of the extract components and increased mass transfer speed. However, the effect of temperature is less than that of pressure because carotenoids are large molecules and their vapor pressure is low [40,41]. Due to the significance of the interaction of pressure and temperature (X_1_X_2_) and quadratic term of pressure (X_1_^2^), the observed increase in capsorubin content with increasing pressure and temperature had a slightly different character than that of other carotenoids. The impact of temperature on the capsorubin extraction is not the same at various pressure. The highest increase in capsorubin content is observed in the high-pressure region (35–45 MPa) at temperatures ranging from 50 °C to 60 °C.

As can be seen in Figure 1f–j, the content of carotenoids in the paprika extract also increases with increasing time at a constant pressure. This is because increasing the time has a positive effect on the chance of sample. It can be caused by the time required for the solvent to penetrate the sample, dissolve the extract and diffuse out of the material. The effect of the extraction time on the content of violaxanthin, capsanthin, and zeaxanthin was similar, and due to the significance of the quadratic term of time, the relationship was non-linear. A significantly greater increase in the content of these carotenoids was observed when the extraction time was in the range of 30–60 min. The increase in the content of carotenoids in the paprika extract as a result of increasing the extraction time depended on the pressure and some differences can be seen in the extraction of capsorubin and β-cryptoxanthin. For capsorubin, the conjugation of higher pressure with time is particularly favorable (Figure 1g). The increase in extraction pressure to 45 MPa resulted in a rapid increase in the content of capsorubin with extraction time. The opposite tendency can be observed in the case of β-cryptoxanthin content (Figure 1j). For the extractions carried out at a pressure of 45 MPa, the content of β-cryptoxanthin slightly increased with increased extraction time. Additionally, in the experiments in which the extraction time was 60 min, a slight increase in its content was noted with increased extraction pressure.

The optimal extraction parameters for maximizing the content of violaxanthin and capsanthin in the paprika extract are as follows: pressure of 45 MPa, temperature of 60 °C, and time of 60 min. To maximize the content of capsorubin, zeaxanthin, and β-cryptoxanthin in the paprika extract, the extraction parameters should be kept in the following range: pressure 42–45 MPa, temperature 45–60 °C, and time 52–60 min.

### 2.2. The Extraction of Carotenoids with Co-Extractant

In this study, SC-CO_2_ with ethanol as a co-extractant for paprika extraction was used at different operating parameters. Co-extractant is added to the solid particle before extraction to improve solute transport properties [26]. The polar modifier molecules accelerate desorption processes by competing with extracts for the active binding sites [38]. It is an innovation towards the current SC-CO_2_ technology by modifying the matrix structure before the extraction process. At the same time, ethanol is a safe solvent for human consumption and is used as a solvent for natural substances for both food and natural medicinal purposes. Table 4 is shown the yield and individual carotenoid content in the paprika extract obtained using the SC-CO_2_ with co-extractant.

The yield of paprika extraction using SC-CO_2_ with co-extractant was higher than that obtained for pure SC-CO_2_ and slightly higher than that of n-hexane (10.84 g/100 g DM). It is because ethanol can increase the polarity of a supercritical fluid mixture even with a small amount added and improve the solubility of the extract components in the supercritical fluid. Moreover, it causes the swelling effect of the solid paprika matrix, thus increasing the internal volume and the contact surface with SC-CO_2_. This in turn results in an increase in the amount of extracted components [26,42].

On the other hand, experimental data denote a negative impact of ethanol as a co-extractant on the carotenoid content in the paprika extract. The addition of ethanol decreased the content of β-carotene and β-cryptoxanthin in the extract and also the content of capsorubin, capsanthin, zeaxanthin and violaxanthin obtained at high pressure. It must be due to the higher affinity of the modified supercritical carbon dioxide to other compounds of non-carotenoid nature. It is known that red paprika carotenoids exhibit some polarity and are high-molecular-weight molecules [41]. The presence of ethanol facilitates their extraction since it can aid the dissolution of heavier substances in CO_2_. However, because of the rich chemical composition of the matrix, the decision of tuning solvent polarity with a modifier can sometimes lead to the dissolution of other compounds, obstructing thus the objective of maximizing only the concentration of target molecules in the final extract [43]. Similarly, Jarén-Galán et al. reported that the yield of paprika extraction using more polar solvents was higher, but the total content of carotenoids was lower [39].

**Figure 2:**
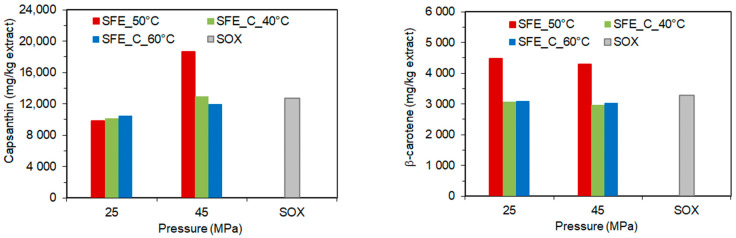
The content of capsanthin and β-carotene in the paprika extract as a function of pressure, temperature, and co-extractant.

It was observed that for SC-CO_2_ extraction with co-extractant increasing pressure increased the content of capsanthin, capsorubin, violaxanthin, and zeaxanthin, but the pressure did not have a significant effect on the content of β-carotene and β-cryptoxanthin in the extract. At the same time, the increasing temperature had only a small negative effect on the content of capsanthin and capsorubin in the extract obtained at high pressure (Table 4). As can be seen (Figure 2), the maximum capsanthin content in the extract obtained in pure SC-CO_2_ at 45 MPa was higher than the maximum capsanthin content achieved with a co-extractant, while the highest β-carotene content in the extract was achieved in pure SC-CO_2_ at 25 MPa.

### 2.3. The Extraction of Fatty Acids

The extraction of fatty acids is receiving increased interest due to their health benefits. Therefore, the effect of the extraction method and condition on the content of fatty acids in the paprika extract was also studied. New data was generated on the fatty acid content of the paprika extract, which is needed due to the lack of information in the literature. The fatty acids composition of the paprika extract obtained using the SC-CO_2_ extraction, extraction with co-extractant, and n-hexane extraction shows in Table 5.

The content of fatty acids in the paprika extracts obtained with SC-CO_2_ ranged from 58.94 to 75.31 g/100 g extract depending on the extraction parameters applied. It was observed that a higher temperature (60 °C) favored the fatty acid content at lower pressure (25 MPa). Moreover, a longer extraction time causes higher fatty acid yield but lower content of fatty acids in the paprika extract. The highest content of fatty acids was achieved for the extract obtained at 25 MPa/60 °C/35 min (SFE4). However, the highest fatty acid yield (63.38 mg/g raw material) was obtained at 35 MPa/60 °C/60 min. Extract with the lowest content of fatty acids and the lowest fatty acid yield was obtained at 45 MPa/50 °C/60 min (SFE5) and 25 MPa/50 °C/10 min (SFE12), respectively.

The content of fatty acids in extracts obtained for SC-CO_2_ extraction with a co-extractant range from 74.28 to 82.64 g/100 g of extract. The maximum fatty acid content for SC-CO_2_ with co-extractant was 1.10-fold higher than the maximum fatty acid content for pure SC-CO_2_ extraction and 1.22-fold higher than that obtained by the Soxhlet method. The addition of ethanol as a co-extractant not only increased the content of fatty acids in obtained extracts but also the fatty acid yield. The maximum fatty acid yield was 1.26-fold higher than in pure SC-CO_2_ extraction. This could be due to the swollen effect when the co-extractant is added to the solid paprika matrix which helps fatty acids to be easily transported out of the solid matrix and facilitated through the SC-CO_2_ solvent bulk. Another reason would be that the co-extractant changed the transport properties of the solute and at the same time acted as a co-solvent to increase the strength of the SC-CO_2_ solvent [26]. The highest fatty acid yield (79.92 mg/g raw material) was found with co-extractant at 45 MPa/60 °C. It was observed that for SC-CO_2_ extraction with a co-extractant, a higher temperature (60 °C) favored the fatty acid content in paprika extract and fatty acid yield at a higher pressure (45 MPa). Ortega et al. also reported that the operating temperature and the addition of pure ethanol as a modifier in SC-CO_2_ enhance the amount of FAs in the extract [44]. They found that the addition of ethanol increases the solubility of the substances including fatty acid compounds. This co-solvent is expected to interact strongly with the target substances via dipole–dipole, hydrogen-bonding, and/or other polarity interactions which may result in a significant enhancement in the extraction yield [45]. The content of fatty acids in the extract obtained by hexane was lower than that by SC-CO_2_ at 25 MPa/60 °C/35 min and similar to that by SC-CO_2_ at 45 MPa/60 °C/35 min.

The majority of fatty acids in the paprika extract were polyunsaturated fatty acids (PUFA), followed by saturated fatty acids (SFA), and monounsaturated fatty acids (MUFA). The PUFA ranged from 40.40 g/100 g of extract for SC-CO_2_ extraction to 57.99 g/100 g of extract for extraction with a co-extractant. An evaluation of the percentage of PUFA of the total FAs was in the range of values from 68.20% to 71.50%. The SFA in paprika extract ranged from 11.31 g/100 g (pure SC-CO_2_ extraction) to 15.23 g/100 g (extraction with co-extractant). The percentage of SFA in the total amount of FAs ranged from 17.89% to 19.90%. The MUFA in paprika extract ranged from 6.94 g/100 g (pure SC-CO_2_ extraction) to 9.42 g/100 g (extraction with co-extractant), and the percentage of MUFA in the total amount of FAs ranged from 11.02% to 12.34%. The results showed that the extraction method and conditions had an effect on the content of fatty acids in the extract, while the proportions of individual fatty acids in the total fatty acid content did not change significantly. The proportion of UFA reported by Chouaibi et al. [18], Embaby et al. [20], and Tepić et al. [21] was slightly higher than those obtained in the present study (83.26–85.49%, 86.50%, 87.14–88.37% of total fatty acids, respectively).

In all of the methods, palmitic acid, stearic acid, lauric acid, arachidic acid, and behenic acid were found as SFA, linoleic acid and α-linolenic acid were found as PUFA, and oleic acid was the only MUFA. The predominant fatty acid of all samples is linoleic acid, followed by palmitic and oleic acids. This finding is comparable with those in the literature [18,21]. Linoleic acid accounts for approximately 80% of the total UFA content in the paprika extract (63.89–66.75% of total fatty acids). However, the identified palmitic acid was the most abundant SFA extracted in all tested conditions, comprising around 80% of the total SFA content. It also shows that the amount of palmitic acid in paprika extract was moderate (9.04–11.79 g/100 g extract).

It is difficult to compare the content of fatty acids in the obtained extract with the literature data. Although there are some studies of SC-CO_2_ extraction of fatty acids from paprika in the literature, the majority just reported SC-CO_2_ extraction of paprika seeds and expressed the fatty acid composition as a percentage of individual fatty acids to total fatty acids. The proportions of linoleic acid in this study (63.89–66.75% of total fatty acids) were lower than those reported by Embaby et al. [20], Azabou et al. [4], Cvetković et al. [46] and Chouaibi et al. [18] for paprika seed oils obtained by SC-CO_2_ (69.40%, 70.93%, 74.01–76.01%, and 76.26% of total fatty acids, respectively) and those given by Tepić et al. [21] for paprika extract (77.20–78.26%), while Abbeddou et al. [47] reported the lowest amount of linoleic acid in paprika oleoresin (55.97%) and a similar amount of α-linolenic acid (5.11%). The amount of α-linolenic acid was lower in other studies. Moreover, Abbeddou et al. [47] and Embaby et al. [20] published slightly higher values for oleic acid (13.81% and 14.4% of total fatty acids, respectively). These discrepancies in the results may be associated with differences in the chemical composition of the raw material used for the study and in the adopted analytical techniques.

**Figure 3:**
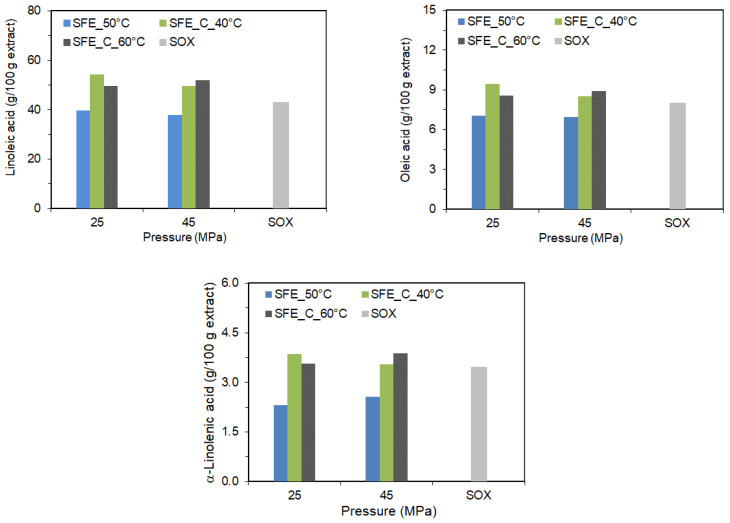
The content of fatty acids in the extract as a function of pressure, temperature, and co-extractant.

As can be seen (Figure 3), the effect of pressure, temperature, and the addition of co-extractant on the content of fatty acids in the extract is completely different than in the case of carotenoids. It was observed that for pure SC-CO_2_ extraction at 50 °C, increasing pressure had only a small negative effect on the linoleic acid content in the extract and a small positive effect on the content of α-linolenic acid. The pressure did not significantly affect the content of other fatty acids. Whereas for SC-CO_2_ extraction with co-extractant at 40 °C, the pressure increase from 25 MPa to 45 MPa had a negative effect on the linoleic, α-linolenic, and oleic acid content of the extracts. Although pressure has improved the extraction yield, a less concentrated fatty acids extract was obtained since the other components were diluted in the product. For the pressure of 25 MPa, the temperature increase from 40 °C to 60 °C also negatively affected the linoleic, α-linolenic, and oleic acid content of the extracts. At the pressure of 45 MPa, the opposite effect was observed. The highest content of linoleic and oleic acid in the paprika extract was obtained at 40 °C/25 MPa with the co-extractant, while the extract with the highest content of α-linolenic acid was obtained with co-extractant at 40 °C/25 MPa and 60 °C/45 MPa, not being statistically different.

Finally, the PUFA/SFA ratios were also calculated and are presented in Table 5. Products with a high PUFA/SFA ratio are considered beneficial for health [4,48], especially because they contribute to the reduction in fat body and total cholesterol [4,49]. Furthermore, PUFA, especially omega-3, when incorporated into the cells can act by modulating several metabolic and signaling pathways and by exerting protective effects against inflammatory and tumoral events. The fatty acid profiles found in the present study were favorable, as the PUFA/SFA ratios of paprika extract were between 4.02 and 4.85, much higher than the recommended. The mean ratio of PUFA/SFA recommended by the British Department of Health is more than 0.45. Meanwhile, WHO/FAO experts suggested a PUFA/SFA ratio above 0.4 in the guidelines for a balanced diet [50]. The PUFA/SFA ratios for all extracts were high due to the elevated amounts of polyunsaturated acids, particularly linoleic acid. According to our results, the red paprika extract may be a valuable source of essential fatty acids, which might be very useful to the cosmeceutical and pharmaceutical industries.

## 3. Material and Methods

### 3.1. Plant Material

Commercial ground red paprika *Capsicum annuum* L. was purchased from P.H. Royal Sp. z o.o. company (Warszawa, Poland). The color value of 160 ASTA (American Spice Trade Association color value) was determined using the spectrophotometric method. The moisture content of 10.35 ± 0.25% was determined using the laboratory moisture analyzer (RADWAG^®^, Poland, model MAC 50/1). The sieve analysis was carried out using the laboratory sieve shaker (Multiserw—Morek Company, Marcyporęba, Poland, model LPzE-2e) and showed that the particle size of the raw material was <0.5 mm.

### 3.2. Techniques of Extraction

Organic solvent extraction was performed in a Soxhlet apparatus. Ground paprika (30 g) was extracted with 300 mL of n-hexane until the complete discoloration of the sample was achieved (4 h). At the end of extraction, the solvent was recovered by rotary evaporation under reduced pressure (Rotavapor R-210, Labortechnik AG Bȕchi, Flawil, Switzerland) at 40 °C. Organic solvent of analytical quality was purchased from Avantor Performance Materials Poland S.A. (Gliwice, Poland). The weight of the obtained extract was gravimetrically measured using the balance (RADWAG®, Radom, Poland, model WPS 4000/C/2) with an accuracy of ±0.1 g. The extract was stored at 4 °C until the analysis was carried out. The extraction was triplicated.

Supercritical carbon dioxide extraction (SFE-CO_2_) processes were performed in the SITEC unit (SITEC-Sieber Engineering AG, Maur (Zurich), Switzerland) previously described in detail [34] with some variations:

Method I: Ground paprika (200 g) was placed in the extractor. The effect of pressure, temperature, and time on SC-CO_2_ extraction of carotenoids was tested by a Box-Benkhen design. Three repetitions were performed at the central design point to estimate the reproducibility of the experimental results. The three levels of temperature: 40, 50, and 60 °C, pressure: 25, 35, and 45 MPa, and time: 10, 35, and 60 min were applied. The extracts were analyzed using HPLC and GC as described.

Method II: Ethanol addition as a co-extractant (raw material/co-extractant mass ratio of 200/75–0.5% relative to the used CO_2_) was added to plant material placed in the extractor and the SC-CO_2_ extraction was performed. The extraction temperature was 40 and 60 °C and the pressure was 25 and 45 MPa. The dynamic extraction time was 60 min.

The CO_2_ flow rate in all SFE experiments was kept at a constant value of 15 kg/h to allow comparison of the obtained results. CO_2_ of 99.9% purity (Grupa Azoty Zaklady Azotowe “Puławy” S.A., Puławy, Poland) was used for the extraction of paprika extract.

### 3.3. Carotenoids Identification and Quantification

Carotenoids were determined in a saponified sample of paprika extract using the internal standard by HPLC analysis. This methodology was described in the previous paper [51]. After saponification, carotenoids were analyzed using a Waters Liquid chromatograph equipped with DAD 2996 detector. Separation of carotenoids was carried out using 4.6 × 150 mm chromatographic column YMC^TM^ Carotenoid with granulation of 3 μm additionally equipped with a protective column YMC^TM^ Carotenoid S-3. The mobile phase was a mixture of methanol, acetonitrile, and water (75:10:15, *v*:*v*:*v*) in gradient elution with dichloromethane. The flow rate was 1 mL/min and the column temperature was set at 25 °C. The injection volume was 20 μL.

Capsanthin of 96% purity, capsorubin of 98% purity, β-carotene of 99% purity, β-cryptoxanthin of 97% purity, violaxanthin of 95% purity, and zeaxanthin of 97% purity standards purchased from CaroteNature GmbH (Münsingen, Switzerland) were used for identification and quantification based on a calibration curve. β-Apo-8′-carotenal of ≥96% purity obtained from Sigma-Aldrich (Buchs, Switzerland) was used as internal standards. The qualitative analysis was based on a comparison of the retention time of peak with an appropriate carotenoid standard.

### 3.4. Gas Chromatography of Fatty Acids

The fatty acid (FA) content in the extract obtained from paprika using n-hexane, pure SC-CO_2,_ and with co-extractant was determined after conversion of the FA to their corresponding methyl esters as described in the previous paper [51]. Samples of extracts were prepared according to ISO standards [52,53,54,55].

Gas chromatography analysis were performed on Agilent equipment (GC 6890N) coupled with mass spectrometer MSD 5975 and equipped with capillary column HP-88 (60 m, 0.25 mm i.d., 0.20 μm film thickness). Measurements were carried out for the following operating parameters of the gas chromatograph: scan range: 50–500 amu; quadrupole temperature: 150 °C; ion source temperature: 230 °C; electron energy: −70 eV. Helium was used as a carrier gas, with a flow rate from 1.0 to 1.5 mL/min. The injection volume was 1 μL.

FAME were identified by comparing their mass spectrum and fragmentation patterns in the NIST library and by comparing retention time peaks with the respective standard. Quantitation analysis was based on analytical standards obtained from Sigma-Aldrich and external calibration curves for each compound separately. The obtained FAMEs value were converted to FA by molecular weight and dilution factor. The FA contents were expressed as g FA/100 g of extract.

### 3.5. Statistical Analysis

The effects of temperature, pressure, and time on the SC-CO_2_ extraction were analyzed by Design Expert (Version 9.0.6.2, Stat-Ease Inc., Minneapolis, MN, USA). Analysis of variance (ANOVA) of the obtained results was performed to evaluate the main three effects (temperature, pressure, and time) and the interaction effects between them. The multiple regression equation, the estimated response surface plot, and the optimum combination of extraction factors were reported for the carotenoid in the paprika extract.

The regression coefficient of determination R^2^, adjusted coefficient of determination R^2^, predicted coefficient R^2^, and lack of fit value were determined to evaluate the fitness of second-order polynomial equation to the response variables. Response surface plots were generated to explain the effects of the independent variables on the response variable. The significance level of all the terms of the second-order polynomial equation was analyzed statistically by computing the *p*-value based on a confidence level of 95%.

## 4. Conclusions

In this work, the effect of different methods (pure SC-CO_2_, SC-CO_2_ with co-extractant, n-hexane) and operating parameters on the extraction of high-value compounds from red paprika *Capsicum annuum* L. was investigated. Results highlighted that the extraction methods significantly affect the yield and content of carotenoids and fatty acids in the paprika extract. It was found that the extraction yield increased from 7.22 to 11.26 g extract/100 g dry weight of sample in pure SC-CO_2_ > n-hexane > SC-CO_2_ with co-extractant order. SC-CO_2_ showed better results in the extraction of carotenoids, but SC-CO_2_ with ethanol as a co-extractant extracted more PUFA than pure SC-CO_2_ and n-hexane.

The results also show that the operating extractive conditions have to be accurately chosen to obtain the highest values of yield and content of compounds. The highest extraction yield and fatty acid yield were obtained at 45 MPa/60 °C/60 min using SC-CO_2_ with co-extractant. Then, the highest content of α-linolenic acid in the extract was found. However, the product rich in oleic and linoleic acid was obtained with co-extractant at 25 MPa/40 °C/60 min. On the other hand, the highest capsanthin, capsorubin, zeaxanthin, β-cryptoxanthin, and violaxanthin content in the extract was obtained using pure SC-CO_2_ at 45 MPa/50 °C/60 min, while the highest β-carotene content was found in the extract obtained at 35 MPa/60 °C/10 min because longer extraction time and higher pressure had a negative effect on the content of β-carotene in the extract.

Results confirmed that the application of SC-CO_2_ in paprika extract production leads to products rich in carotenoids and polyunsaturated fatty acids that can be used as valuable products in the pharmaceutical, cosmetic, and food industries. It is possible to regulate the content of valuable components by adjusting the operating parameters of the SC-CO_2_ extraction process.

## Figures and Tables

**Table 1:** The carotenoid content in the paprika extract.

Run Order	Extraction Parameters			Y (g/100 g DM)	Content of Carotenoids (mg/kg Extract)					
	p, MPa (X_1_)	T, °C (X_2_)	t, min (X_3_)		Violaxanthin	Capsorubin	Capsanthin	Zeaxanthin	β-Cryptoxanthin	β-Carotene
SFE1	35 (0)	60 (1)	60 (1)	9.94	450 ± 18.1	2250 ± 113.1	13,860 ± 494.3	1050 ± 45.8	860 ± 5.9	4330 ± 262.5
SFE2	35 (0)	50 (0)	35 (0)	8.75	150 ± 2.8	1300 ± 39.3	7650 ± 37.7	640 ± 0.4	690 ± 3.5	3680 ± 6.1
SFE3	35 (0)	50 (0)	35 (0)	8.85	280 ± 9.9	1230 ± 25.6	7950 ± 5.7	620 ± 2.9	680 ± 9.0	3720 ± 23.9
SFE4	25 (−1)	60 (1)	35 (0)	8.68	160 ± 1.2	620 ± 25.8	4370 ± 132.7	420 ± 14.7	660 ± 13.3	4250 ± 133.7
SFE5	45 (1)	50 (0)	60 (1)	9.50	750 ± 6.7	3070 ± 108.0	18,610 ± 24.3	1380 ± 13.5	970 ± 0.6	4300 ± 75.7
SFE6	25 (−1)	40 (−1)	35 (0)	8.35	60 ± 2.2	640 ± 36.3	3930 ± 16.4	320 ± 1.4	410 ± 5.8	3410 ± 79.5
SFE7	35 (0)	40 (−1)	10 (−1)	8.04	170 ± 0.6	730 ± 5.3	4950 ± 43.1	370 ± 3.2	440 ± 3.0	3470 ± 21.2
SFE8	35 (0)	40 (−1)	60 (1)	8.93	340 ± 1.4	1910 ± 21.1	11,430 ± 166.2	860 ± 0.9	770 ± 4.6	3800 ± 9.5
SFE9	25 (−1)	50 (0)	60 (1)	9.11	340 ± 4.0	1400 ± 3.0	9810 ± 264.8	780 ± 3.3	830 ± 1.3	4480 ± 27.1
SFE10	45 (1)	60 (1)	35 (0)	9.48	500 ± 7.2	2500 ± 16.9	14,030 ± 101.7	1070 ± 15.8	790 ± 4.8	3630 ± 38.4
SFE11	45 (1)	50 (0)	10 (−1)	8.35	320 ± 5.1	1330 ± 19.9	8640 ± 37.0	690 ± 3.5	760 ± 1.5	4540 ± 4.1
SFE12	25 (−1)	50 (0)	10 (−1)	7.22	n.d.	920 ± 83.2	1720 ± 133.1	140 ± 0.9	260 ± 1.0	3060 ± 23.5
SFE13	35 (0)	60 (1)	10 (−1)	8.09	240 ± 3.2	990 ± 37.1	6640 ± 78.9	570 ± 8.3	810 ± 12.8	4970 ± 63.0
SFE14	35 (0)	50 (0)	35 (0)	8.76	270 ± 19.6	1320 ± 32.3	7490 ± 141.9	650 ± 21.4	790 ± 10.6	4650 ± 127.4
SFE15	45 (1)	40 (−1)	35 (0)	8.61	390 ± 10.0	1900 ±2.6	12,130 ± 92.7	880 ± 21.3	780 ± 8.3	3820 ± 41.4
SOX				10.84	610 ± 9.5	2020 ± 50.3	13,270 ± 268.7	1000 ± 12.4	740 ± 14.8	3290 ± 60.5

SFE1–SFE15: SC-CO_2_ extraction; SOX: Soxhlet extraction with n-hexane; n.d.—not detectable. Data are given as the mean values ± SD, number of replicates *n* = 3.

**Table 2:** The regression coefficients of full quadratic models with their statistical significance and model parameters for carotenoids.

Source	Violaxanthin (mg/kg)		Capsorubin (mg/kg)		Capsanthin (mg/kg)		Zeaxanthin (mg/kg)		β-Cryptoxanthin (mg/kg)	
	β	*p*-Value	β	*p*-Value	β	*p*-Value	β	*p*-Value	β	*p*-Value
Model		0.0149		0.0011		0.0003		0.0003		0.0195
Lack of Fit		0.4740		0.0440		0.0469		0.0379		0.3009
β_0_	233.33		1283.30		7696.67		636.67		720.00	
X_1_	175.00	0.0014	652.50	0.0001	4197.50	<0.0001	295.00	<0.0001	142.50	0.0048
X_2_	48.75	0.1354	147.50	0.0616	807.50	0.0404	85.00	0.0107	90.00	0.0285
X_3_	143.75	0.0033	582.50	0.0002	3970.00	<0.0001	287.50	<0.0001	145.00	0.0044
X_1_X_2_	2.50	0.9511	155.00	0.1345	365.00	0.4200	22.50	0.4912	−60.00	0.2101
X_1_X_3_	22.50	0.5867	315.00	0.0151	470.00	0.3094	12.50	0.6971	−90.00	0.0836
X_2_X_3_	10.00	0.8067	20.00	0.8271	185.00	0.6748	−2.50	0.9375	−70.00	0.1544
X_1_^2^	48.33	0.2845	170.83	0.1175	696.67	0.1682	35.42	0.3125	−37.50	0.4275
X_2_^2^	−4.17	0.9217	−39.17	0.6830	221.67	0.6301	0.42	0.9900	−22.50	0.6266
X_3_^2^	70.83	0.1395	225.83	0.0547	1301.67	0.0298	75.42	0.0623	22.50	0.6266
R^2^		0.9384		0.9791		0.9879		0.9874		0.9311
Adjusted R^2^		0.8275		0.9415		0.9661		0.9648		0.8072
Predicted R^2^		0.3096		0.6744		0.8113		0.8032		0.0991

*p* < 0.0001 very highly significant, *p* < 0.01 very significant, and *p* > 0.10 not significant.

**Table 3:** The regression coefficients of reduced mathematical models with their statistical significance and model parameters for response variables.

Source	Violaxanthin (mg/kg)		Capsorubin (mg/kg)		Capsanthin (mg/kg)		Zeaxanthin (mg/kg)		β-Cryptoxanthin (mg/kg)	
	β	*p*-Value	β	*p*-Value	β	*p*-Value	β	*p*-Value	β	*p*-Value
Model		<0.0001		<0.0001		<0.0001		<0.0001		0.0005
Lack of Fit		0.6889		0.0691		0.0617		0.0707		0.3283
β_0_	258.57		1259.33		8221.43		657.14		700.00	
X_1_	175.00	<0.0001	652.50	<0.0001	4197.50	<0.0001	259.00	<0.0001	142.5	0.0010
X_2_	48.75	0.0576	147.50	0.0275	807.50	0.0205	85.00	0.0008	90.00	0.0164
X_3_	143.75	<0.0001	582.50	<0.0001	3970.00	<0.0001	287.50	<0.0001	145.00	0.0009
X_1_X_2_			155.00	0.0782						
X_1_X_3_			315.00	0.0041					−90.00	0.0691
X_2_X_3_										
X_1_^2^			173.90	0.0611						
X_2_^2^										
X_3_^2^	67.68	0.0694	228.90	0.0219	1236.1	0.0165	72.86	0.0195		
R^2^		0.9152		0.9781		0.9758		0.9824		0.8456
Adjusted R^2^		0.8813		0.9562		0.9661		0.9754		0.7838
Predicted R^2^		0.8129		0.7825		0.9410		0.9560		0.6149

*p* < 0.0001 very highly significant, *p* < 0.01 very significant, and *p* > 0.10 not significant.

**Table 4:** Results of paprika extraction using SC-CO_2_ with ethanol as co-extractant.

SFE Condition	Y (g/100 g DM)	Content of Carotenoids (mg/kg Extract)					
		Violaxanthin	Capsorubin	Capsanthin	Zeaxanthin	β-Cryptoxanthin	β-Carotene
25 MPa/40 °C	10.37	370 ± 0.1	1620 ± 9.6	10,130 ± 151.5	740 ± 6.7	650 ± 10.9	3060 ± 9.7
25 MPa/60 °C	10.71	380 ± 8.6	1610 ± 39.3	10,490 ± 249.1	800 ± 0.8	660 ± 18.5	3090 ± 17.3
45 MPa/40 °C	11.04	540 ± 0.1	1960 ± 6.5	12,900 ± 267.3	940 ± 17.7	700 ± 12.7	2970 ± 75.0
45 MPa/60 °C	11.26	540 ± 6.1	1810 ± 23.4	11,920 ± 183.9	910 ± 16.2	670 ± 8.9	3030 ± 29.9

Data are given as the mean values ± SD; number of replicates *n* = 3.

**Table 5:** Fatty acid content in extracts obtained from paprika *Capsicum annuum* L. by different methods.

	Content of Fatty Acids (g/100 g Extract)											PUFA/SFA	Total FA Yield (mg/g Sample)
	Lauric Acid (C12:0)	Palmitic Acid (C16:0)	Stearic Acid (C18:0)	Oleic Acid (C18:1)	Linoleic Acid (C18:2)	Arachidic Acid (C20:0)	α-Linolenic Acid (C18:3)	Behenic Acid (C22:0)	Total SFA	Total UFA	Total FA		
SFE1	0.64 ± 0.03	11.09 ± 0.16	1.98 ± 0.06	8.48 ± 0.02	45.55 ± 0.28	0.27 ± 0.01	3.07 ± 0.03	0.21 ± 0.00	14.19	57.10	71.29	3.43	63.38
SFE2	0.47 ± 0.09	11.11 ± 0.04	2.01 ± 0.02	8.64 ± 0.02	46.87 ± 0.14	0.27 ± 0.00	3.03 ± 0.01	0.21 ± 0.00	14.07	58.54	72.61	3.55	56.85
SFE3	0.49 ± 0.02	10.14 ± 0.75	1.79 ± 0.14	7.89 ± 0.53	42.59 ± 3.16	0.29 ± 0.01	2.69 ± 0.26	0.11 ± 0.06	12.82	53.17	65.99	3.53	52.26
SFE4	0.40 ± 0.02	11.55 ± 0.06	2.11 ± 0.01	9.08 ± 0.07	48.53 ± 0.49	0.27 ± 0.01	3.16 ± 0.11	0.21 ± 0.01	14.54	60.77	75.31	3.56	58.44
SFE5	0.34 ± 0.01	9.04 ± 0.97	1.58 ± 0.25	6.94 ± 0.86	37.84 ± 4.15	0.21 ± 0.01	2.56 ± 0.39	0.14 ± 0.05	11.31	47.34	58.65	3.57	49.85
SFE6	0.30 ± 0.05	10.54 ± 0.96	1.91 ± 0.22	8.27 ± 0.74	44.72 ± 3.68	0.27 ± 0.00	2.76 ± 0.28	0.17 ± 0.05	13.19	55.75	68.94	3.60	51.50
SFE7	0.31 ± 0.04	11.66 ± 0.17	1.38 ± 0.04	8.16 ± 0.30	49.13 ± 1.03	0.29 ± 0.01	3.03 ± 0.17	0.12 ± 0.08	13.76	60.32	74.08	3.79	53.26
SFE8	0.50 ± 0.05	10.94 ± 0.62	1.96 ± 0.12	8.50 ± 0.49	45.64 ± 2.61	0.27 ± 0.00	3.03 ± 0.23	0.14 ± 0.10	13.81	57.17	70.98	3.52	56.71
SFE9	0.30 ± 0.03	9.18 ± 0.29	1.59 ± 0.08	7.01 ± 0.19	39.62 ± 1.65	0.21 ± 0.01	2.31 ± 0.06	0.11 ± 0.05	11.39	48.94	60.33	3.68	49.17
SFE10	0.48 ± 0.03	10.47 ± 0.04	1.81 ± 0.04	8.02 ± 0.01	43.48 ± 0.06	0.24 ± 0.03	2.89 ± 0.07	0.17 ± 0.04	13.17	54.39	67.56	3.52	57.29
SFE11	0.41 ± 0.01	10.34 ± 0.27	1.83 ± 0.04	7.96 ± 0.27	43.47 ± 1.06	0.28 ± 0.01	2.67 ± 0.06	0.14 ± 0.00	13.00	54.10	67.10	3.55	50.12
SFE12	0.20 ± 0.01	11.30 ± 0.29	2.02 ± 0.12	8.76 ± 0.39	47.74 ± 2.00	0.27 ± 0.01	2.91 ± 0.15	0.17 ± 0.05	13.96	59.41	73.37	3.63	47.40
SFE13	0.39 ± 0.07	11.08 ± 0.68	1.99 ± 0.14	8.60 ± 0.47	46.88 ± 2.62	0.29 ± 0.01	2.92 ± 0.18	0.15 ± 0.01	13.90	58.40	72.30	3.58	52.35
SFE14	0.53 ± 0.02	11.22 ± 020	2.07 ± 0.05	9.10 ± 0.11	47.37 ± 0.85	0.29 ± 0.01	2.93 ± 0.11	0.22 ± 0.01	14.33	59.40	73.73	3.51	57.80
SFE15	0.63 ± 0.05	10.60 ± 0.91	1.85 ± 0.17	8.05 ± 0.74	43.65 ± 3.78	0.25 ± 0.01	2.88 ± 0.35	0.18 ± 0.04	13.51	54.58	68.09	3.44	52.43
SOX	0.78 ± 0.02	10.40 ± 0.34	1.80 ± 0.05	8.02 ± 0.22	43.05 ± 1.15	0.26 ± 0.01	3.45 ± 0.10	0.17 ± 0.05	13.41	54.52	67.93	3.47	65.89
SFE_C_25 MPa/40 °C	0.73 ± 0.03	11.79 ± 0.15	1.84 ± 0.02	9.42 ± 0.12	54.15 ± 0.82	0.55 ± 0.05	3.84 ± 0.07	0.32 ± 0.01	15.23	67.41	82.64	3.91	76.65
SFE_C_25 MPa/60 °C	0.75 ± 0.04	10.23 ± 0.39	1.55 ± 0.07	8.52 ± 0.13	49.43 ± 0.81	0.56 ± 0.03	3.55 ± 0.17	0.31 ± 0.03	13.40	61.50	74.90	3.95	71.75
SFE_C_45 MPa/40 °C	0.87 ± 0.04	9.46 ± 0.75	1.54 ± 0.14	8.48 ± 0.71	49.58 ± 3.63	0.53 ± 0.04	3.53 ± 0.35	0.29 ± 0.01	12.69	61.59	74.28	4.19	73.35
SFE_C_45 MPa/60 °C	0.86 ± 0.06	11.33 ± 0.42	1.73 ± 0.08	8.88 ± 0.02	51.81 ± 0.13	0.55 ± 0.04	3.87 ± 0.28	0.30 ± 0.05	14.77	64.56	79.33	3.77	79.92

FA fatty acids; SFA saturated fatty acids; UFA unsaturated fatty acids; PUFA polyunsaturated fatty acids; SFE1-SFE15: SC-CO_2_ extraction; SOX: Soxhlet extraction with n-hexane; SFE_C: SC-CO_2_ extraction with co-extractant. Data are given as the mean values ± SD; number of replicates *n* = 3.

## Data Availability

All data analyzed during this study are included in this article.

## Sample Availability

Samples of the compounds are available from the authors.
